# How Does Task Presentation Impact Motor Inhibition Performance in Young Children?

**DOI:** 10.3389/fpsyg.2021.684444

**Published:** 2021-08-02

**Authors:** Kathryn Mason, Alice Bowmer, Graham F. Welch

**Affiliations:** UCL Institute of Education, University College London, London, United Kingdom

**Keywords:** young children, task presentation, assessment, executive function, motor skills, inhibition, peg tapping

## Abstract

Peg tapping tasks are commonly used as a measure of inhibitory skill in young children. However, differences in the way the task is presented may influence children’s performance. For example, if a peg tapping task is presented at regular intervals, children can entrain to the presentation pulse, which may in turn support their performance. This study assessed how speed and regularity of presentation may support or impair children’s responses. An experimenter was filmed delivering the tapping task at two different speeds (120 bpm/3,000 ms per trial and 150 bpm/2,400 ms per trial). Additionally, they were filmed delivering the task at regular intervals (i.e., the onset of each trial was predictable), or at irregular intervals (the onset of each trial was unpredictable). *N* = 103 children aged between 5 and 6 years old were tested on the task. They completed one block with 20 regular interval trials and another block with 20 irregular interval trials. Block presentation order was randomized. Children who achieved over 90% accuracy on the task were then presented with two more blocks at 150 bpm. Children’s response accuracy was measured. Our results show a difference in children’s accuracy across all conditions with trials presented in an irregular manner producing poorer performance on the task. The study demonstrates how speed and regularity of presentation can affect children’s scores on a tapping task used to measure inhibition. Demands on working memory, motor ability, and speed of processing are all affected by adjustments in presentation. Entrainment to a pulse is also a potential mechanism employed by children to support their performance on this task.

## Introduction

During the 1960s and 1970s, eminent neuropsychologist Alexander Luria carried out many experiments examining the function of the frontal lobes – the brain region that synthesizes information about the outside world, regulates behavior in coordination with action, and is highly involved in motor control and language (Chayer and Freedman, 2001). From these experiments, Luria et al. (1964) published one particular case study of a patient with left frontal lobe lesions during which they analyzed the patient’s responses to a series of simple tasks involving both spoken and motoric responses. One of these assessments continues to be widely used today, and is known as the peg tapping task.

Commonly used in clinical settings, the peg tapping task involves an experimenter tapping on a table, after giving the patient the instruction –*“when I tap once, you tap twice.”* Patients with frontal lobe damage find it very difficult to inhibit a prepotent response – in this instance, the urge to copy the experimenter rather than follow the verbal instruction. Luria referred to this motoric repetition as an “echopractic” response, but after lengthy practice and training, Luria’s patient was able to produce the correct response (i.e., tapping twice when the experimenter tapped once). However, at the introduction of a second opposing rule –*“when I tap twice, you tap once,”* the patient was unable to sustain the correct performance and reverted back to echopractic responses. Luria noted that patients with damage to the frontal lobes often have difficulty taking on two rules at once, despite their ability to recall each rule individually (Barbizet, 1970; Luria, 1973).

Luria and colleagues devised the peg tapping task as one of multiple assessments that required the ability to hold two things in mind simultaneously (working memory), to alternate between one rule and another (cognitive flexibility), and to suppress a prepotent behavior or action in favor of another (to exercise inhibitory control over one’s behavior). The task continues to be used widely in clinical settings, as well as more recently in developmental and behavioral studies with children as a measure of complex response inhibition.

The history of psychological research into inhibition reveals a cyclical pattern, with a resurgence in interest recurring every 20 to 25 years. Interestingly the term “Inhibition” began to be applied in research on list learning during the early to mid 20th Century (Melton and Von Lackum, 1941). At a similar time Luria and Vygotsky were exploring the concept experimentally, using a variety of different terms to describe inhibition responses. However, little more appears to be published in this area (Dempster and Corkill, 1999) until it resurfaces in the fields of developmental and educational psychology a few decades later where it is considered in relation to the developing inhibition skills of young children (Passler et al., 1985). Several key steps in the study of inhibition were achieved by the development and implementation of the Go/No Go task (Donders, 1969) and the no-stop signal task (Logan et al., 1984), variations of which are commonly used to measure inhibitory skill in a range of populations.

Today, inhibition is described as one of the three core executive functions (EFs), along with working memory and cognitive flexibility, (Miyake et al., 2000), with selective attention. The development of complex EFs and their individual trajectories have been of particular interest to developmental psychologists, educators, and those who work in fields linked to child development (see Best and Miller, 2010, for a review). The publication of various cognitive and neurophysiological assessments have recently allowed researchers to chart the development of these component skills from the first few years of life into adulthood, with significant gains in EF skill seen throughout childhood and adolescence.

Inhibitory control is a highly complex executive function, implemented by an extensive network of brain regions including the frontal and parietal lobes, and several basal ganglia (Mirabella, 2014). However, a distinction can be made between motor and interference components of inhibition (Wostmann et al., 2013). Here, motor inhibition identifies the ability to inhibit a pre-planned motor response whilst in contrast, interference inhibition is the ability to resolve conflicting response options in order to perform appropriately (Mirabella, 2021). It is suggested that the peg tapping task measures a form of interference inhibition.

As seen with the Peg tapping task, EF measures that have been adapted from well-established adult tasks present multiple complications for valid and reliable assessment in younger age groups. This is at least partially due to young children’s comparatively limited attentional duration (Simpson and Riggs, 2005), attentional control (e.g., increased susceptibility to distraction; Best et al., 2009; Howard et al., 2014), and ability to understand instructions and communicate their response (Hughes, 1998a). Measurement is made even more complex by the fact that inhibitory skills (e.g., waiting your turn, or being patient) develop over the course of childhood, gradually improving as children mature.

A variety of tasks have been developed in order to assess young children’s complex inhibition skills, for example, Flanker Tasks (e.g., Diamond et al., 2007) the Hearts and Flowers task (Diamond and Wright, 2014), the Sun/Apple task (Simon, 1990), the Hand Game (Hughes, 1998b), the Day/Night task (Gerstadt et al., 1994), and more recently, a computerized version of the stop-signal task, suitable for use with 6 year old children (Mirabella et al., 2020). In the hand game (Luria et al., 1964; Hughes, 1998b), which is similar to the peg tapping task, children must produce a closed fist when shown a flat palm by the experimenter and *vice versa*. This task eliminates the requirement for verbal instruction or use of numbers, however, it does require fast gestural responses that may be too difficult for many preschoolers.

Alternatively, the Day/Night Task (Gerstadt et al., 1994) involves the inhibition of verbal responses, instead of the motoric responses found in the peg tapping task and hand games. Here, children are shown an image of the sun and asked to say “night” as their response, and when they are shown an image of the moon, they should respond by saying “day.” Sometimes a control condition is included as a precursor, where children have to copy the experimenter rather than inhibit their impulse to copy and following this, the experimenter then presents the conflict (test) condition. This introduces a switching element to the task, whereby the children have to inhibit a previous response (rule; Garon et al., 2008).

Diamond and Taylor (1996) developed the peg tapping task for use with children aged 3–7 years. In their longitudinal study, they predicted that they would see an increasing improvement in children’s performance on the task over time, as children’s ability to hold two things in mind and inhibit prepotent responses improve markedly between 3 and 6 years of age. In the 25 years since Diamond and Taylor’s publication, the peg tapping task has been widely used in developmental research as a measure of motor inhibitory skill in young children. Most researchers use it as a straightforward, two rule inhibition task, presented live by the experimenter, with some authors also reporting the latency of presentation by measuring the length of time between experimenter delivery and child response.

Previous studies have provided details about their procedures for administering the peg tapping task (Diamond and Taylor, 1996; Hala et al., 2003; Domitrovich et al., 2007; Bierman et al., 2008). However, there are currently limited guidelines regarding the speed at which trials are presented across the task, and experimenters are likely to have different natural speeds of presentation both of which confound the comparison of results across children and studies. This becomes further complicated in studies where the experimenter and child share one dowel for tapping.

Diamond and Taylor ensured that experimenters were carefully trained so that they did not influence children’s responses; such as, for example, not reaching for the dowel too quickly, or pausing for too long after the child’s response. However, there are currently no published studies which examine the components of the peg tapping task, or the potential issues that arise due to it being presented live. Although, several studies discuss a variety of issues surrounding the adaptation and presentation of other EF tasks. Most of these studies are concerned with converting live performance tasks into computerized versions, which allow more control of trial presentation and time response limits.

One way of controlling presentation variation is to deliver a task via computer, or using an electronic tablet. Here, the validity of converting a task from live to computerized presentation, as well as their differences, have been tested experimentally in many recent publications – for example, Brunetti et al. (2014) and Björngrim et al. (2019). Brunetti et al. (2014), looked at the differences between the traditional Corsi Block Tapping Task and eCorsi in which they found the latter to control for inter-stimulus presentation timings, whilst also emphasizing additional benefits in ease of administration (because of task automation), as well as precise scoring and measurement of reaction times. They note that the temporal accuracy of manual tapping is particularly difficult to control by the experimenter, who can (inadvertently) be slower or faster depending on multiple factors. In live administration set-ups, psychologists have anecdotally complained that when administering particularly long sequences they are forced to slow down the pace of block tapping. This is because they have to read the sequence in order to remember it, however, most studies do not report on these factors (Brunetti et al., 2014).

Additionally, we observe that live experimenter influence in the peg tapping task has been overlooked. In our previous study (Bowmer et al., 2018), we used the peg tapping task to measure children’s inhibition and we noticed inter-individual entrainment between experimenter and some children during task presentation. It made us aware of how open this task was to experimenter effects. For example, the regularity of trials, or presenting the task at a slightly different speed, could help or hinder children, depending on how developed their inhibitory skills were. To further examine our observations, we used the peg tapping task in two ways: as a measure of children’s inhibitory skill, and to compare children’s performance after we manipulated two different parameters of the task; speed and regularity of presentation. For clarity, the current study was also part of a wider project investigating executive function skills in primary school children.

The current study aimed to answer three questions. The first of these is:

Q1: How does regularity of task presentation affect children’s performance on Luria’s peg tapping task?

Our 2-tailed hypothesis included the following potential outcomes:

a) If regular presentation produces better accuracy, it is an indication that predictability supports performance on an inhibition task.
b) If irregular presentation produces better accuracy, it is an indication that regularity interferes with performance on an inhibition task.

Null hypothesis: There is no difference in accuracy between conditions: regular or irregular presentation has no impact on performance on an inhibition task.

The second and third questions explore other key variables:

Q2: How do children perform at a faster speed?
Q3: Finally, is there an interaction between regularity and speed? I.e., does a faster speed help or hinder children’s accuracy during regular and irregular presentation?

## Methodology

### Creation of the Peg Tapping Task

To address the research questions, two aspects of the peg tapping task were manipulated; the regularity (spacing of trials) and speed of trials.

Two different versions of the task were created (versions A and B) adapted from the instrument documentation made available by The Peabody Institute: https://my.vanderbilt.edu/cogselfregulation/files/2012/12/Peg-Tapping-without-stats-info.pdf. In version A, trials were presented at regular intervals, and in version B, trials were presented at irregular intervals. In the regular version, the onset of each trial was spaced evenly (every 6 beats). In the irregular condition, trials were presented at irregular intervals, so that the onset of each trial was unpredictable, but still delivered within a 6-beat framework. Trials were presented in the same order for each version (see appendix). Validity and reliability for this task have been conducted by (Blair and Razza, 2007) and (Lipsey et al., 2017).

The experimenter was filmed presenting the trials for version A and B at a speed of 120 bpm/3,000 ms per trial (hereafter referred to as 120 bpm), and each version was saved as an individual video. They were then filmed presenting versions A and B at the faster speed of 150 bpm/2,400 ms per trail (hereafter referred to as 150 bpm). All videos were approximately 1 min in duration. Details of all versions are available as Supplementary Material.

In this experimental set-up we chose to create video of a person administering the task rather than using a fully-computerized version in order to maintain a level of similarity to the in-person peg-tapping task, whilst controlling certain elements of presentation (speed and regularity).

### Participants

*N* = 103 children between the ages of 5 years 6 months and 6 years 4 months (mean age 5 years 9 months) were recruited for the study from three Primary schools in London. There were 52 males and 51 female children. The parents/carers provided informed written consent in advance for their children to participate, as did the participant schools. Ethical approval for the study was provided by the UCL IoE Research Ethics Committee under data protection number No Z6364106/2019/01/85 social research, 18th January 2019.

### Procedure

Children were randomly assigned to receive either task version A or version B first within each school. Half of the children received version A first, and the other half started with version B.

The experimenter and child participant sat next to one another, each equipped with a pencil. It is important to note that in the current study the child was always in control of their tapping pencil, as the experimenter presented trials via a screen instead of live. A laptop showing the videos was set up in front of the participant. The experimenter explained the rules of the peg tapping game to the child participant and each rule was practiced after instruction. The instructions were as follows: “When I tap once, I want you to tap twice. Let’s have a go now.” “Ok, good. Now, when I tap twice, I want you to tap once. Have a go. Good. Are you ready to play the game?” The participant was then given four pre-trials with the experimenter, administered live, to check that they understood the instructions. If the participant made errors on any of the pre-trials, the experimenter repeated the rules and a further practice trial was given. If the participant continued to make errors, the experimenter moved to a copycat version of the task. Participants were then presented with 20 trials of the peg tap task (A or B) in a pseudo-random order via a pre-recorded person on the computer screen. The experimenter recorded children’s responses and scored the task live. If the children could not follow the rule, they continued with the task, but only had to copy the presented trials. If children were unable to follow the task rules they received a score of 0.

**FIGURE 1:**
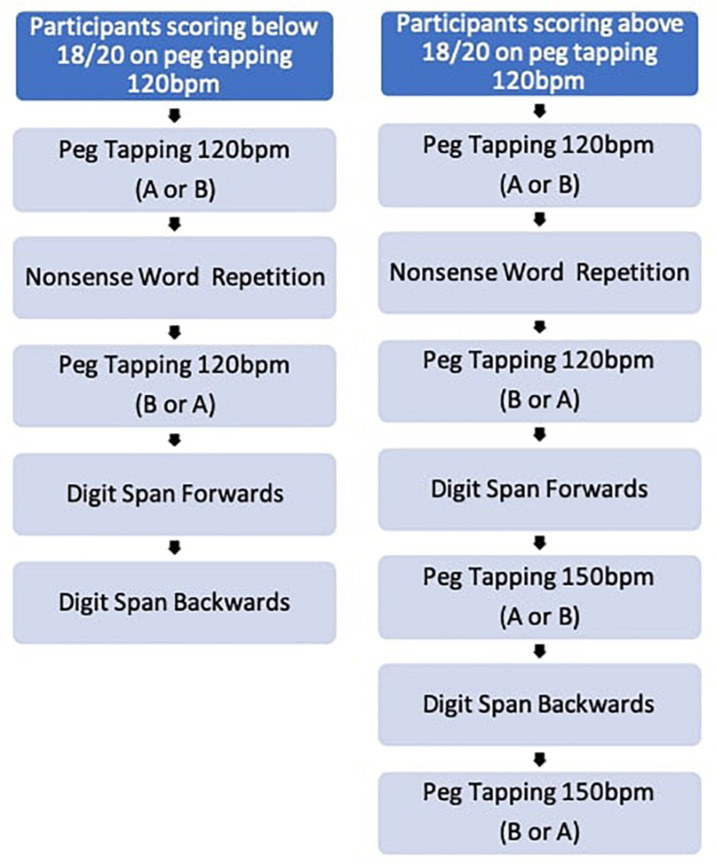
Task procedure.

In order to avoid fatigue and to give children a break between different versions of the task, children were also presented with a non-word repetition task (Gathercole et al., 1994) and a digit span task (Wechsler and Kodama, 1949). See Figure 1 for the order of task presentation.

Children who scored above 18 out of 20 on both peg tapping tasks delivered at 120 bpm were asked to play the game at a faster speed of 150 bpm, followed by the Backwards Digit Span task. The testing procedure concluded with the alternate version of Peg tapping task (B or A). See Figure 1.

## Results

### Q1: Does Presentation Regularity Impact Participant Performance?

Children completed the task at 120 bpm in both presentation conditions (regular and irregular). One child did not complete both tapping tasks, so her data were excluded from the detailed analyses. The mean scores for each condition are shown in Table 1 below. Despite being able to do the task during the practice trials, seven children copied the experimenter during the task, and could not follow the rules. Therefore, they were given a score of 0.

**TABLE 1:** Mean scores and SD for the task presented at 120 bpm.

*N* (Average age)	Regular 120 bpm	Irregular 120 bpm	*t*
102 children (5.9 months)	16.73 (SD 5.20)	15.59 (SD 4.94)	*t*_(__101__)_ = 4.776, *p* < 0.001

A paired samples *t*-test showed that the difference in children’s task performance between the two conditions was significant [*t*_(__101__)_ = 4.776, *p* < 0.001]. Therefore, regular trial presentation appears to support children’s performance on the tapping task, whilst irregular trial presentation was more challenging.

### Q2: How do Children Perform at a Faster Speed?

Of the 102 children who completed the task at 120 bpm, *n* = 31 children performed at or near ceiling on the task (i.e., a score of 18 or higher in both presentation conditions). Those children were given the task again, this time presented at 150 bpm. The same testing procedure was followed, with initial presentation condition randomized between participants. Their mean scores for each presentation condition are presented in Table 2 below.

**TABLE 2:** Mean scores and SD for the task presented at 150 bpm.

*N*	Regular 150 bpm	Irregular 150 bpm	*t*
31 children (5.8 months)	18.90 (SD 1.22)	17.06 (SD 2.54)	*t*_(__30__)_ = 4.350, *p* < 0.001

A paired samples *t*-test showed that the difference in children’s task performance between the two conditions was significant [*t*_(__30__)_ = 4.350, *p* < 0.001]. Therefore, the same pattern of results is found at a faster speed, with children performing significantly more accurately in the regular condition than the irregular condition.

### Q3: Does a Faster Speed Help or Hinder Children’s Accuracy During Regular and Irregular Presentation?

Our third research question examined the interaction between regularity and speed. Data from the 31 children who completed the task in all conditions was analyzed to investigate this question. A repeated measures ANOVA found a significant difference in children’s performance across all four conditions of speed and regularity *F*_(__3_,_90__)_ = 19.3, *p* < 0.001. A *post hoc* Tukey test showed that there were significant differences in children’s performance between the regular 120 bpm condition and the irregular 150 bpm condition (*t* = 7.183, *p* < 0.001); the 120 bpm irregular and 150 bpm regular condition [*t*_(__90__)_ = 5.547, *p* < 0.001] and between the regular and irregular conditions at 150 bpm [*t*_(__90__)_ = 5.183, *p* < 0.001] see Table 3.

**TABLE 3:** *Post hoc* Tukey *t*-test comparing differences in performance across all conditions.

Condition comparison		Mean difference	*t*	*P*(Tukey)
120 bpm regular	120 bpm irregular	0.581	1.637	0.363
	150 bpm regular	0.710	2.000	0.196
	150 bpm irregular	2.548	7.183	<0.001
120 bpm irregular	150 bpm regular	0.129	0.364	0.983
	150 bpm irregular	1.968	5.547	<0.001
150 bpm regular	150 bpm irregular	1.839	5.183	<0.001

**FIGURE 2:**
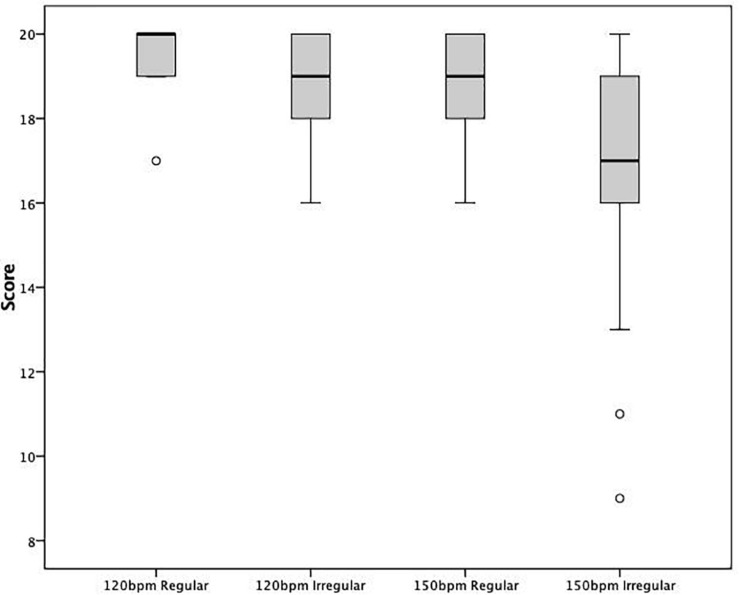
The distribution of scores across all conditions of the peg tapping task

Box plots (shown in Figure 2) illustrate the distribution of scores on the peg tapping task across all conditions. The plots show a wider range of scores on the task when it is presented at a faster speed and with irregular presentation.

At 120 bpm, 10 children scored full marks in both conditions. However, at 150 bpm only 3 children achieved a maximum score in both conditions. Table 4 shows the number of children who achieved the maximum score of 20 in each of the conditions. Significantly fewer children achieved the maximum score in both the regular and irregular conditions at 150 bpm. An analysis of variance revealed significant differences in the maximum scores achieved between the 120 bpm regular and irregular conditions [*t*(93) = 3.450, *p* = 0.001] and the 120 bpm regular and 150 bpm regular conditions [*t*(30) = 2.187, *p* = 0.037].

**TABLE 4:** Number of participants achieving maximum score in each condition.

*N* = 102		*N* = 31	
120 bpm regular	120 bpm irregular	150 bpm regular	150 bpm irregular
31	15	12	5

## Discussion

The current study aimed to investigate how task presentation might impact motor inhibition performance in young children. This was tested experimentally by controlling and manipulating the peg tapping task to assess how different conditions affect scores. Our results show that the speed and regularity of trial presentation both had a significant effect on children’s accuracy.

It is notable that when the task was presented at 120 bpm, 15 children achieved a maximum score in the 120 bpm irregular condition, but at 150 bpm, this was the case for only 5 of these same children. Children who were good at the task and scored highly in both regular and irregular conditions at 120 bpm were not able to maintain this result at a faster speed in the irregular presentation condition, despite extensive practice on the task. The irregular condition at 150 bpm was the most challenging condition for participants.

The results support our first hypothesis that regular presentation results in better accuracy scores, and faster presentation results in poorer performance (regardless of presentation regularity). Children performed significantly better when the trials were delivered at predictable intervals, rather than unpredictable intervals. Children also performed significantly better when the task was presented at a speed of 120 bpm opposed to a faster speed of 150 bpm. These two findings suggest that predictable trial onsets support children’s performance, and that faster task presentation at 150 bpm hinders their performance at this age. At both 120 bpm and 150 bpm, unpredictable trial onsets had a negative effect on children’s accuracy when compared to the predictable onset condition. These findings are important as they highlight the potential for experimenter influence on child performance. Even small variations between experimenters could potentially influence between-participant results.

A key challenge in assessing executive function skills in young children is the predisposition for administrators to adjust their presentation style depending on the perceived capabilities of the participant in front of them. For example, if a child appears to be able to do the task well, the experimenter may inadvertently speed up the presentation of the trials in order to challenge or hold their attention. Likewise, if a child is struggling with the task, the tendency may be for the experimenter to slow down to allow them more time for success or to hold their attention. Whilst differences in presentation may be very subtle and unintentional, variation in test presentation could be problematic when looking across children’s performance at a group level. Researchers should ensure, as far as possible, that children are all receiving a task of comparable difficulty in the test situation. In studies using computerized tasks, a staircase procedure is sometimes implemented in order to determine a participant’s threshold of ability on a given task. This resolves the issue of maintaining challenge whilst staying within a participant’s ability level in a controlled way (e.g., Mirabella et al., 2020).

In live cognition testing, researchers have long been aware of experimenter effects (Sattler and Theye, 1967; Overton et al., 2016). However, this has not been explored for the peg tapping task which is often used in clinical settings to determine patients inhibitory skills, or as an indication of potential neurological problems. When the peg tapping task was originally published for use with children, Diamond and Taylor (1996) measured children’s response latency between the time the experimenter finished tapping to the point at which the child made their response. They noted that response latencies decreased over the course of the testing session, with children taking a longer time to respond on the first four trials and becoming faster on subsequent trials as the testing session went on. Equally, they observed that accuracy decreased as the testing session progressed, with children making more errors on later trials than on earlier ones (children try harder at the beginning).

Diamond and Taylor reflect on the possible reasons for this pattern of findings (which they found replicated when they administered the Day/Night task) and suggest that cognitive fatigue and loss of attention as the testing session progressed to be some of the possible causes. They postulate that a faster response time, coupled with fewer correct responses as the task progressed was indicative of a lack of ability to sustain attention, and therefore the children were more likely to respond haphazardly rather than make considered attempts at responding correctly. Arguably, this is exactly the time that an experimenter is likely to adjust their response. More conscious of the two-way interactional nature of this task, (Diamond and Taylor, 1996) were careful to avoid cuing the child for the right response by taking or passing the dowel too quickly, but they did not address formally the possibility that the experimenter would have any influence on task speed.

Whilst Diamond and Taylor do report response latency between experimenter onset and child response, they do not record the latency between children’s responses and the experimenter’s tap on the next trial. This is an important part of the picture because it shows the pattern of flow between trials as well as who might be driving the speed of trial onset and period. It is possible that the increase in speed in later trials could be partially driven by experimenter effects, with the experimenter “speeding up” their presentation of trials, either as an attempt to engage children’s attention, or to bring the task to an end. Likewise, longer latency in earlier trials could be due to an experimenter’s natural tendency to establish the children’s ability level on the task, subsequently speeding up their presentation rate on later trials, which the child then “keeps up” with.

While loss of attention may contribute to some children’s poorer performance, this study also questioned whether regular task presentation could impede children’s concentration through entrainment. In this case, children’s responses become more automated, rather than conscious and effortful, and they stop paying attention to the responses they are giving. This may be true for children who achieved high accuracy on the task. If the prepotent response is no longer to copy, but to respond with the relevant number of taps (which has become entrained), the task is no longer a measurement of children’s inhibitory skill.

One of the limitations of the current study was the use of a pre-recorded video of the experimenter administering the task. Whilst this may take from the ecological validity of the study, it was necessary in order to control the speed and predictability of taps in each of the conditions. Earlier piloting involving a click-track being played in the experimenter’s ear proved to be too difficult to maintain during live administration.

When children are presented with the peg tapping task, it is often in the context of a “game.” Live presentation of the task makes it a “two-player game” between the experimenter and the child. However, when the task is presented via computer or pre-recorded video, it becomes a “one-player” game, with the child responding to stimuli, but not interacting with another person. To date, the peg tapping task has been delivered mostly in a live presentation format which allows for experimenter and child interaction. In this circumstance, the experimenter may also give extra time or explanation to support performance, whereas if delivered in a highly controlled environment less support can be provided. Both of these circumstances present trade-offs; increased experimental control is gained through computer-based tasks; however, some children will perform better when interacting with a real person. In this study, we wanted to control the parameters of the task to see how live presentation may be affecting child performance, as this had not been done before.

Findings from this study are relevant for all research investigating children’s inhibitory skills using the peg tapping task, as the findings suggest that outcomes are open to experimenter effects in both speed and regularity of presentation. This also applies to clinical environments with adult participants and patients and may also be true for other cognitive tasks that involve face-to-face assessment. Further research with different age groups and larger numbers of children in all conditions could both confirm and increase evidence for these experimenter effects. These effects can be solved by using computerized tasks, however, this in turn removes some of the real-world relevance, raising questions about how applicable findings are to children’s everyday experiences.

## Data Availability Statement

The datasets presented in this article are not readily available because participants did not agree for their data to be shared publicly. Requests to access the datasets should be directed to the corresponding author.

## Ethics Statement

Ethical approval for the study was provided by the UCL IOE Research Ethics Committee under data protection number No Z6364106/2019/01/85 social research, 18th January 2019.

## Author Contributions

KM, AB, and GW designed the research. KM and AB collected and analyzed the data. KM and AB prepared and wrote the manuscript with support from GW. All authors contributed to the article and approved the submitted version.

## Conflict of Interest

The authors declare that the research was conducted in the absence of any commercial or financial relationships that could be construed as a potential conflict of interest.

## Publisher’s Note

All claims expressed in this article are solely those of the authors and do not necessarily represent those of their affiliated organizations, or those of the publisher, the editors and the reviewers. Any product that may be evaluated in this article, or claim that may be made by its manufacturer, is not guaranteed or endorsed by the publisher.
