# Supplementary material for: How Does Task Presentation Impact Motor Inhibition Performance in Young Children?
Source: Front Psychol. 2021 Aug 2;12:684444. doi: 10.3389/fpsyg.2021.684444 (PMC8366059; doi:10.3389/fpsyg.2021.684444)
Supplement: Supplementary file 1 [file Table_1.DOCX]

6. Supplementary Materials.

Order of taps in the regular condition

| Trial | Number of taps | Beats per trial |
| --- | --- | --- |
| 1 | 2 | (6) |
| 2 | 1 | (6) |
| 3 | 2 | (6) |
| 4 | 1 | (6) |
| 5 | 2 | (6) |
| 6 | 2 | (6) |
| 7 | 1 | (6) |
| 8 | 1 | (6) |
| 9 | 1 | (6) |
| 10 | 2 | (6) |
| 11 | 1 | (6) |
| 12 | 2 | (6) |
| 13 | 2 | (6) |
| 14 | 1 | (6) |
| 15 | 1 | (6) |
| 16 | 2 | (6) |
| 17 | 1 | (6) |
| 18 | 2 | (6) |
| 19 | 2 | (6) |
| 20 | 1 |  |

Order of taps in the irregular condition

| Trial | Number of taps | Beats per trial |
| --- | --- | --- |
| 1 | 2 | (5) |
| 2 | 1 | (7) |
| 3 | 2 | (8) |
| 4 | 1 | (6) |
| 5 | 2 | (5) |
| 6 | 2 | (6) |
| 7 | 1 | (8) |
| 8 | 1 | (4) |
| 9 | 1 | (5) |
| 10 | 2 | (5) |
| 11 | 1 | (5) |
| 12 | 2 | (8) |
| 13 | 2 | (7) |
| 14 | 1 | (4) |
| 15 | 1 | (8) |
| 16 | 2 | (5) |
| 17 | 1 | (5) |
| 18 | 2 | (9) |
| 19 | 2 | (5) |
| 20 | 1 |  |
